# Impact of Systemic Inflammatory Response Syndrome on Clinical, Echocardiographic, and Computed Tomographic Outcomes Among Patients Undergoing Transcatheter Aortic Valve Implantation

**DOI:** 10.3389/fcvm.2021.746774

**Published:** 2022-02-09

**Authors:** Tarek A. N. Ahmed, You-Jeong Ki, You-Jung Choi, Heba M. El-Naggar, Jeehoon Kang, Jung-Kyu Han, Han-Mo Yang, Kyung Woo Park, Hyun-Jae Kang, Bon-Kwon Koo, Hyo-Soo Kim

**Affiliations:** ^1^Department of Internal Medicine, Cardiovascular Center, Seoul National University Hospital, Seoul, South Korea; ^2^Department of Cardiovascular Medicine, Assiut University Heart Hospital, Assiut, Egypt

**Keywords:** systemic inflammatory response syndrome, hypoattenuation leaflet thickening, transcatheter aortic valve implantation, subclinical leaflet thrombosis, inflammatory markers

## Abstract

**Background:**

Systemic inflammatory response syndrome (SIRS) is a systemic insult that has been described with many interventional cardiac procedures. The outcomes of patients undergoing transcatheter aortic valve implantation (TAVI) are thought to be influenced by this syndrome not only on short-term, but also on long-term.

**Objective:**

We assessed the association of SIRS to different clinical, echocardiographic, and computed tomographic (CT) outcomes after TAVI.

**Methods:**

Two hundred and twenty-four consecutive patients undergoing TAVI were enrolled in this study. They were assessed for the occurrence of SIRS within the first 48 h after TAVI. Patients were followed-up for short- and long-term clinical outcomes. Serial echocardiographic follow-ups were conducted at 1-week, 6-months, and 1-year. CT follow-up at 1 year was recorded.

**Results:**

Eighty patients (36%) developed SIRS. Among different parameters, only pre-TAVI total leucocytic count (TLC), pre-TAVI heart rate, and post-TAVI systolic blood pressure independently predicted the occurrence of SIRS. The incidence of HALT was not significantly different between both groups, albeit higher among SIRS patients (*p* = 0.1) at 1-year CT follow-up. Both groups had similar patterns of LV recovery on serial echocardiography. Long-term follow-up showed that all-cause death, cardiac death, and re-admission for heart failure (HF) or acute coronary syndrome (ACS) were significantly more frequent among SIRS patients. Early safety and clinical efficacy outcomes were more frequently encountered in the SIRS group, while device-related events and time-related valve safety were comparable.

**Conclusion:**

Although SIRS implies an early acute inflammatory status post-TAVI, yet its clinical sequelae seem to extend to long-term clinical outcomes.

## Introduction

The outcome of patients undergoing surgical or interventional therapy is unfavorably influenced by severe systemic inflammation. Systemic inflammatory response syndrome (SIRS) has been reported to frequently occur in the setting of TAVI. Its exact mechanism is still unknown. Periprocedural hypotension and suboptimal organ perfusion with ischemia-reperfusion injury and subsequent cytokine release have been suspected to play a role in the pathogenesis of SIRS post-TAVI (1, 2).

A previous study has accused the inflammatory process post-TAVI to the decline in LV function recovery (3). Likewise, inflammation have been suggested as a contributor to the pathogenesis of acute and chronic heart failure (4).

Hypoattenuation leaflet opacities on computed tomography (CT), or the so called hypoattenuation leaflet thickening (HALT) have been reported after TAVI. It is a hallmark finding of subclinical leaflet thrombosis (5), and have provoked a debate about the necessity of routine anticoagulation post-TAVI, with safety and efficacy concerns. An inflammatory hypothesis has been implicated in the provocation of HALT (6).

Despite SIRS is reported in the early post-TAVI phase, yet its clinical impact has been evidenced to extend to the long-term outcomes with the earlier valve generations (7). Thus, we hypothesize that a sort of a long-standing inflammatory milieu might implicate this finding and is early explicated as SIRS.

In this study, we attempted to elucidate the impact of SIRS on the echocardiographic, CT, and short- and long-term VARC-2-defined individual and composite outcomes, among a real-world population with variable risk scores and utilizing earlier and latest generations transcatheter aortic valves.

## Materials and Methods

### Patient Population

We retrospectively included all patients with severe AS (indexed aortic valve area ≤0.6 cm^2^/m^2^ or transvalvular mean gradient >40 mm Hg or peak velocity >4 m/s) treated with trans-catheter aortic valve implantation (TAVI) in the period between 2012 till 2019 at Seoul National University Hospital. Patients were divided into 2 groups according to the development of SIRS. All medical records were systematically reviewed and retrieved for patient medical history, vital status, diagnostic tests including lab values, echocardiographic and CT angiographic data as well as procedural details. Patients' clinical data was reported up to the latest follow-up from the hospital electronic medical records. Patients were excluded if they were converted to open heart surgery as the surgical trauma and extracorporeal circulation exacerbate the inflammatory response (8–10), or if they died within <48 h after TAVI, which precludes the feasibility of SIRS data assessment. Part of the study population was also included in the K-TAVI registry previously published (11). The study complied with the declaration of Helsinki and was approved by the local ethics committee.

### Clinical Endpoints

SIRS was defined, according to the existing ACCP/SCCM consensus guidelines, as fulfilling at least two of the following four criteria within the first 48 h after TAVI as assessed by electronic monitoring records in the intensive care unit (ICU): temperature <36.0 or >38.0°C, heart rate >90 beats/min, hyperventilation as respiratory rate >20 breaths/min or P_a_CO_2_ <32 mmHg, leucocytic count >12 or <4 (10^9^/L) (12, 13) (Supplementary Table 1). Clinical outcomes were assessed in accordance with the standardized end-point definitions of the Valve Academic Research Consortium 2 (VARC-2) (14).

### Echocardiographic Analysis

Trans-thoracic echocardiographic studies were performed for all participants at baseline before TAVI, then follow-up was performed at 1-week, 1-month, and 1-year post-TAVI. Follow-up was available in 78 (97.5%), 143 (99.3%) patients at 1-week, 66 (82.5%), 132 (92%) at 1 month, and 47 (59%), 117 (81%) at 1 year, among the SIRS and no SIRS groups, respectively. Measurements included AV study (aortic valve area (AVA), and mean pressure gradient), left ventricular ejection fraction (LVEF) determined by 2-D biplane Simpson's method, left ventricular mass index (LVMI), left atrial volume index (LAVI), tissue doppler assessment of mitral annulus for estimation of S', E', and E/E' ratio, as well as 2-D LV global longitudinal strain (2-D LV-GLS) by speckle tracking echocardiography. All measurements were standardized according to latest American Society of Echocardiography guidelines (15), and echocardiographic-related endpoints complied to definitions as per VARC-2 consensus document (14).

### CT Angiographic Analysis

CT analysis was performed routinely before the TAVI procedure for proper planning of valve size, procedure, and vascular access. Post-TAVI CT was performed routinely at 1-year follow-up, according to rigorous institutional protocols, for the assessment of hypoattenuation leaflet thickening (HALT), that often indicate leaflet thrombosis formation (16), as well as measurements of different aortic dimensions. The protocol adopted was standardized throughout the study period and was largely consistent with the latest consensus document by the Society of Cardiovascular Computed Tomography (SCCT) (17). Follow-up CT studies at 1 year were available for 45 (56.3%), and 109 (75.7%) patients among the SIRS and no SIRS groups, respectively.

### Statistical Analysis

Continuous variables were expressed as mean ± standard deviation or median (interquartile range), while categorical variables were presented as frequencies and percentages. Continuous variables were compared by Student's *t*-test or non-parametric tests according to whether normally distributed or not, respectively. Chi-square or Fisher's exact tests were used to compare categorical variables. Repeated measures ANOVA was used to compare the within-groups and between-groups effect of repeated echocardiographic measurements over time and to assess group-time interaction. Logistic regression modeling was used to assess the independent predictors of SIRS among different covariates. Cox regression analysis was performed to assess the cumulative events over time among the study groups. Two models were created; one for overall mortality and the other for composite clinical efficacy VARC-2 defined endpoints. Variables with *p* < 0.05 on univariate analyses were included in a multivariate model. Survival analysis according to the occurrence of SIRS was determined for each of overall mortality and clinical efficacy using Kaplan-Meier (K-M) method. The log-rank test was used to express statistical difference in survival analysis. Through-out all tests, statistical significance was assumed when two-sided *p* < 0.05. Statistical analyses were performed using SPSS statistics version 21 (IBM Corporation, NY, USA).

## Results

### Baseline Clinical Characteristics and Periprocedural Laboratory Findings

A total of 224 patients were included in our analysis. Of those 80 patients (36%) developed SIRS, while 144 (64%) had no SIRS. Table 1 shows the baseline clinical characteristics of both study groups; those with SIRS vs. those with no SIRS. Both groups were comparable regarding most clinical parameters. However, those with SIRS had significantly higher STS risk score as well as higher baseline heart rate.

**Table 1:** Baseline clinical parameters.

**Parameter**		**SIRS (*n =* 80)**	**No SIRS (*n =* 144)**	***P*-value**
Male gender		38 (47.5%)	75 (52.1%)	0.5
Age		78.3 ± 8.3	77.4 ± 7.2	0.4
BMI		23.5 ± 3.5	23.6 ± 3.4	0.7
DM		36 (45%)	49 (34%)	0.11
Hypertension		59 (73.8%)	107 (74.3%)	0.9
COPD		19 (23.8%)	28 (19.4%)	0.4
Malignancy		7 (8.8%)	18 (12.5)	0.4
PAD		5 (6.3%)	14 (9.7%)	0.4
Prior CABG		1 (1.3%)	4 (2.8%)	0.5
Prior PCI		25 (31.3%)	53 (36.8%)	0.4
Prior MI		3 (3.8%)	10 (6.9%)	0.4
Bicuspid valve		12 (15%)	28 (19.6%)	0.4
EUROSCORE II		4.2 (2.1–7.3)	3.0 (1.8–5.8)	0.05
STS score		5.8 (3.2–14.8)	3.8 (2.4–7.2)	<0.001
Pre-TAVI NYHA FC	NYHA 1–2	40 (50.6%)	86 (60.1%)	0.2
	NYHA 3–4	39 (49.4%)	57 (39.9%)	
CAD	1 VD	13 (16.5%)	21 (14.9%)	0.7
	2 VD	14 (17.7%)	20 (14.2%)	
	3 VD	9 (11.4%)	23 (16.3%)	
	LMD	5 (6.3%)	10 (7.1%)	0.8
Pre-TAVI HR		76 ± 15.7	67 ± 11.8	<0.001
Pre-TAVI AF		11 (13.8%)	14 (9.7%)	0.4
Pre-TAVI 1st degree AVB		6 (7.5%)	12 (8.3%)	0.8
Pre-TAVI RBBB		8 (10%)	9 (6.3%)	0.3
Pre-TAVI LBBB		2 (2.5%)	3 (2.1%)	0.8
Prior CAVB/PPM		2 (2.5%)	3 (2.1%)	0.8

*SIRS, systemic inflammatory response syndrome; BMI, Body mass index; DM, diabetes mellitus; COPD, chronic obstructive pulmonary disease; PAD, peripheral arterial disease; CABG, coronary artery bypass graft; PCI, percutaneous coronary intervention; MI, myocardial infarction; STS, society of thoracic surgeons; NYHA FC, New-York heart association functional classification; CAD, coronary artery disease; VD, vessel disease; TAVI, transcatheter aortic valve implantation; HR, heart rate; AF, atrial fibrillation; AVB, atrioventricular block; RBBB, right bundle branch block; LBBB, left bundle branch block; CAVB, complete atrioventricular block; PPM, permanent pacemaker*.

Table 2 shows the baseline laboratory findings of both study groups. Patients with SIRS had significantly lower baseline hemoglobin levels, while they elicited higher total leucocytic count (TLC), Neutrophil lymphocyte ratio (NLR), absolute neutrophilic count (ANC), procalcitonin, and high-sensitive C-reactive protein (HS-CRP) at baseline.

**Table 2:** Baseline and follow-up laboratory investigations.

**Parameter**	**SIRS (*n =* 80)**	**No SIRS (*n =* 144)**	***P*-value**
**Baseline**			
Hemoglobin	11 ± 2.1	11.7 ± 1.8	0.02
Platelets	194.6 ± 68	191.3 ± 70	0.7
TLC	7.5 ± 3.4	6.2 ± 1.7	<0.001
Neutrophil	63.4 ± 12.6	60 ± 10.6	0.03
Lymphocyte	23.1 ± 11	27.3 ± 9.3	0.003
NLR	2.7 (1.8-4.6)	2.2 (1.6–3.0)	0.009
ANC	5,104 ± 3,280	3,769 ± 1,439	0.005
Serum creatinine	1.7 ± 1.9	1.2 ± 1.1	0.015
Procalcitonin	1.1 (0.3–2.7)	0.11 (0.06–0.23)	0.015
Cystatin C	1.78 ± 1.2	1.41 ± 1	0.11
NT Pro-BNP	1,671 (439–4,379)	813 (344–3,289)	0.12
CK-MB	1.6 (1.1–2.7)	1.5 (1.1–2.5)	0.9
Troponin	0.03 (0.01–0.08)	0.02 (0.01–0.1)	0.9
HS-CRP	0.25 (0.05–1.19)	0.12 (0.05–0.40)	0.02
**Post-procedural**			
Hemoglobin	10.5 ± 1.6	11.0 ± 1.4	0.02
Platelets	165.1 ± 75	151.5 ± 65.7	0.2
TLC (mean 48 h.)	11.9 ± 4.4	8.4 ± 2.1	<0.001
Neutrophil	84.6 ± 6.7	81.3 ± 6.4	<0.001
Lymphocyte	7.3 ± 4.4	10.4 ± 4.5	<0.001
NLR	13.4 (8.7–18.2)	8.8 (6.2–12.2)	<0.001
ANC	11364 ± 4500	7520 ± 2051	<0.001
Serum creatinine	1.6 ± 1.9	1.2 ± 1.1	0.2
Cr Cl	48.3 ± 29.4	54.2 ± 21	0.1
E GFR	66 ± 35	73 ± 28	0.1
Peak CK-MB	13 (7.7–19.4)	9.7 (6.1–15.1)	0.01
Peak troponin	2.7 (1.4–5.1)	1.6 (0.9–3.2)	0.002
Peak HS-CRP	8.1 (4.7–12.3)	3.8 (2.5–5.9)	<0.001

*SIRS, systemic inflammatory response syndrome; TLC, total leucocytic count; NLR, neutrophil lymphocyte ratio; ANC, absolute neutrophilic count; NT Pro-BNP, N terminal pro- brain natriuretic peptide; CK-MB, creatine kinase-myocardial band; HS-CRP, high sensitive C reactive protein; Cr Cl, creatinine clearance*.

Table 2 shows post-procedural laboratory data, which were quite similar to the baseline findings, with significantly higher indices of inflammation as well as myocardial injury as evidenced by CK-MB and troponin.

### Procedural Characteristics

There was a diversity among the valve types and generations utilized throughout the study period, which were similarly distributed between the study groups. There was no significant difference in the procedural parameters between both groups with the exception of post-implantation systolic and diastolic blood pressures which were significantly lower among the SIRS group (Table 3).

**Table 3:** Procedural characteristics.

**Parameter**		**SIRS (*n =* 80)**	**No SIRS (*n =* 144)**	***P*-value**
Valve type	Edwards SAPIEN THV	4 (5%)	4 (2.8%)	0.7
	Edwards SAPIEN XT	5 (6.3%)	4 (2.8%)	
	Edwards SAPIEN 3	24 (30%)	49 (34%)	
	CoreValve	17 (21.3%)	29 (20.1%)	
	Evolut R	18 (22.5%)	41 (28.5%)	
	Evolut Pro	2 (2.5%)	4 (2.8%)	
	Lotus	10 (12.5%)	13 (9%)	
Valve size	23	22 (27.5%)	38 (26.4%)	0.9
	26	34 (42.5%)	58 (40.3%)	
	29	24 (30%)	47 (32.6%)	
	34	0 (0%)	1 (0.7%)	
Vascular access	Transfemoral	77 (96.3%)	142 (98.6%)	0.3
	Transapical	3 (3.8%)	2 (1.4%)	
General anesthesia		78 (97.5%)	137 (95.1%)	0.4
Anesthesia duration (min.)		152.5 ± 60.8	138.1 ± 46.8	0.05
Duration of admission (days)		13 (9.8–20)	10 (9–12)	<0.001
Procedure to discharge duration (days)		10 (8–15)	8 (7–9)	<0.001
Procedure duration (min.)		75 ± 36	72.4 ± 37.5	0.6
Balloon pre-dilatation		28 (35%)	59 (41%)	0.4
Size of pre-dilatation balloon		20.3 ± 1.1	20.6 ± 2	0.5
Balloon post-dilatation		31 (38.8%)	57 (39.6%)	0.9
Size of post-dilatation balloon		23.9 ± 2.7	24.1 ± 1.9	0.7
VIV		2 (2.5%)	6 (4.2%)	0.5
Oversizing Index		14.2 ± 8.3	13.9 ± 7.4	0.8
Pre-TAVI ARI		26.6 ± 11	24.8 ± 10.1	0.3
Post-TAVI ARI		28.2 ± 7.3	27.7 ± 7.5	0.7
Pre-TAVI AV mean PG (mmHg)		54.9 ± 22.7	54.8 ± 21.4	0.9
Post-TAVI AV mean PG (mmHg)		5.7 ± 5.1	6.4 ± 7.3	0.5
Post-implant SBP		125 ± 20	132 ± 21	0.02
Post-implant DBP		66 ± 12	69 ± 14	0.04
Post-implant HR		75 ± 13	73 ± 13	0.2
RCC valve depth (mm)		3.8 ± 3	4.7 ± 3.3	0.1
NCC valve depth (mm)		4 ± 2.6	4.4 ± 3.1	0.5
Residual PVL	No/Trivial	64 (80%)	123 (85.4%)	0.3
	Mild	16 (20%)	19 (13.2%)	
	Moderate	0 (0%)	2 (1.4%)	

*SIRS, systemic inflammatory response syndrome; TAVI, Transcatheter aortic valve implantation; ARI, aortic regurge index; AV, aortic valve; PG, pressure gradient; SBP, systolic blood pressure; DBP, diastolic blood pressure; HR, heart rate; RCC, right coronary cusp; NCC, non-coronary cusp; PVL, para-valvular leakage*.

### CT Angiographic Findings

Table 4 shows the baseline and 1-year follow-up CT data for the study groups. Baseline parameters were not significantly different. The follow-up rate was significantly lower among the SIRS group (56.3 vs. 75.7%, *p* = 0.003). Noticeably, the incidence of HALT, on follow-up CT, was higher among the SIRS group yet not reaching statistical significance (31.1 vs. 20.2%, *p* = 0.1).

**Table 4:** Baseline and 1-year follow-up CT angiographic findings.

**Parameter**	**SIRS (*n =* 80)**	**No SIRS (*n =* 144)**	***P*-value**
**Baseline parameters**			
Annulus perimeter	73.9 ± 7	73.9 ± 9.6	0.9
Annulus area	422.3 ± 93.8	432.8 ± 90	0.4
Perimeter derived annulus diameter	23.5 ± 2.3	24.2 ± 5.6	0.4
Area derived annulus diameter	23.2 ± 2.2	23.2 ± 3.2	0.9
STJ diameter	28 ± 8.4	27.2 ± 4.1	0.4
SOV diameter	30.8 ± 3.3	31.1 ± 3.7	0.6
LVOT diameter	23 ± 2.4	23.5 ± 2.9	0.2
LCA height from annulus	12.6 ± 3.1	12.7 ± 3.2	0.7
RCA height from annulus	14.9 ± 3.5	15.3 ± 3.7	0.4
Proximal AA diameter	36.9 ± 4.6	37.5 ± 4.4	0.3
**1-year follow-up**			
Follow-up rate	45 (56.3%)	109 (75.7%)	0.003
Proximal AA diameter	36.6 ± 4.6	37.5 ± 5.8	0.4
STJ diameter	24.5 ± 5.6	24.3 ± 3.9	0.9
HALT	14 (31.1%)	22 (20.2%)	0.1
Clinical thrombosis^*^	4 (8.9%)	11 (10.1%)	0.8

* *Necessitating anticoagulation*.

*CT, computed tomography; SIRS, systemic inflammatory response syndrome; STJ, Sino-tubular junction; SOV, sinus of Valsalva; LVOT, left ventricular outflow tract; LCA, left coronary artery; RCA, right coronary artery; AA, ascending aorta; HALT, hypoattenuation leaflet thickening*.

### Echocardiographic Data

**Figure 1:**
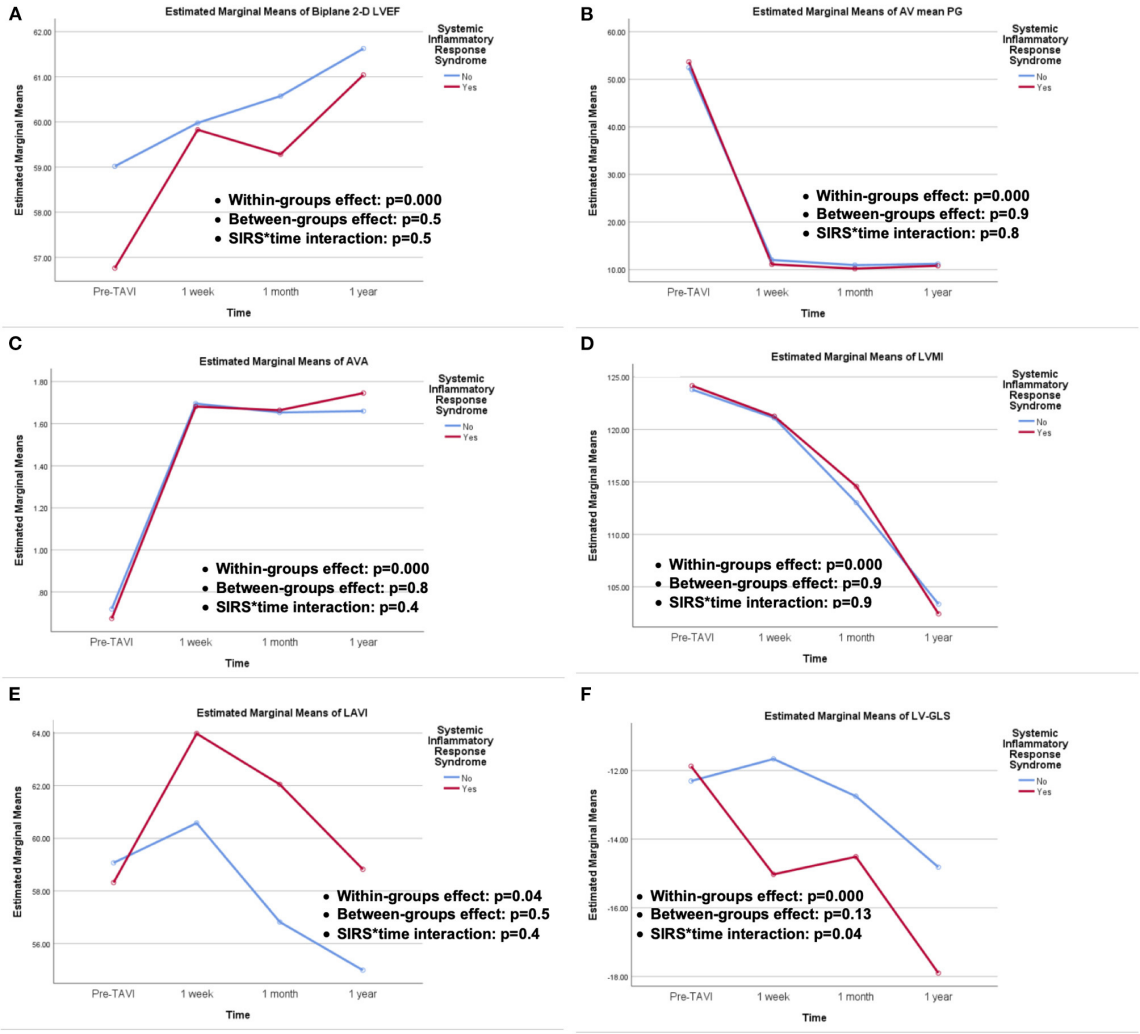
Repeated measures ANOVA for echocardiographic outcomes of; **(A)** 2-D LVEF, **(B)** AV mean PG, **(C)** AVA, **(D)** LVMI, **(E)** LAVI, and **(F)** LV-GLS, showing the within-groups and between-groups effect, as well as SIRS groups and time interactions.

TTE was performed at baseline and at predetermined follow-up points (Table 5). At baseline, no significant difference was encountered among study groups except for higher PASP in the SIRS group, which faded out on follow-up. No significant difference in echocardiographic parameters was encountered upon follow-up at different time intervals. A repeated measure ANOVA was conducted to compare different echocardiographic parameters among the study groups at different time intervals (Figure 1). The within-group difference over time was significant for all echocardiographic parameters; 2D-LVEF, AV mean PG, AVA, LVMI, LAVI, and LV-GLS, while there was no significant difference between both study groups (between-groups effect) for the aforementioned parameters. Regarding the interaction of study groups over time, it was not significant except for LV-GLS which elicited significant interaction with time (*p* = 0.04).

**Table 5:** Baseline and Follow-up echocardiographic findings.

**Parameter**		**SIRS** **(*n =* 80)**	**No SIRS** **(*n =* 144)**	***P*-value**
**Baseline**				
AV peak velocity		4.5 ± 0.8	4.6 ± 0.7	0.5
AV mean gradient		51.7 ± 19.1	52.7 ± 18.3	0.7
AVA		0.69 ± 0.24	0.72 ± 0.18	0.4
AVAI		0.44 ± 0.14	0.45 ± 0.12	0.6
LVIDD		48 ± 6.9	47.8 ± 5.9	0.8
Biplane LVEF		56.5 ± 12.4	59.6 ± 10.3	0.1
LAVI		62.5 ± 28.1	60.6 ± 22.2	0.6
LVMI		128.1 ± 35.1	129.1 ± 36.6	0.9
EE' medial		23.5 ± 9.2	23 ± 12.4	0.8
EE' lateral		18.2 ± 9	18.5 ± 9	0.9
S' medial		4.3 ± 1.4	4.5 ± 1.3	0.2
S' lateral		5.1 ± 1.6	5.4 ± 1.5	0.4
LV 2D GLS		−12.4 ± 3.8	−13.6 ± 3.9	0.15
PASP		45.3 ± 12.3	40.2 ± 9.7	0.003
RWMA		11 (13.8%)	20 (13.9%)	0.9
AR	No/Trivial	54 (67.5%)	94 (65.3%)	0.5
	Mild	18 (22.5%)	40 (27.8%)	
	Moderate	6 (7.5%)	10 (6.9%)	
	Severe	1 (1.3%)	0 (0%)	
MR	No/Trivial	56 (70%)	120 (83.3%)	0.12
	Mild	20 (25%)	20 (13.9%)	
	Moderate	4 (5%)	4 (2.8%)	
MS	No	72 (90%)	130 (90.3%)	0.3
	Mild	5 (6.3%)	4 (2.8%)	
	Moderate	3 (3.8%)	10 (6.9%)	
**1-week post-TAVI**				
AV peak velocity		2.5 ± 1.7	2.4 ± 0.7	0.5
AV mean gradient		10.7 ± 4.2	11.9 ± 5.3	0.1
AVA		1.7 ± 0.5	1.7 ± 0.4	0.9
AVAI		1.09 ± 0.28	1.07 ± 0.27	0.6
LVIDD		47.1 ± 6.7	46.8 ± 6.7	0.7
Biplane LVEF		58.7 ± 10	60.2 ± 8.6	0.3
LAVI		63.2 ± 42	62.1 ± 36.1	0.8
LVMI		123 ± 31.7	124 ± 30	0.8
EE' medial		23.3 ± 8.6	23.3 ± 11.6	0.9
EE' lateral		17.7 ± 7.1	17.9 ± 9.5	0.9
S' medial		5.1 ± 1.3	5.5 ± 1.5	0.1
S' lateral		6.7 ± 1.8	6.5 ± 1.9	0.6
LV 2D GLS		−12.8 ± 3.9	−13.2 ± 3.9	0.6
PASP		39.9 ± 9	37.2 ± 7.2	0.04
Para-valvular AR	No/Trivial	57 (73%)	103 (72.1%)	0.9
	Mild	17 (21.8%)	34 (23.8%)	
	Moderate	4 (5.1%)	6 (4.2%)	
Prosthetic AV stenosis	Normal	68 (95.8%)	136 (98.6%)	0.2
	Mild	3 (4.2%)	2 (1.4%)	
Patient prosthesis mismatch	Insignificant	57 (80.3%)	125 (90.6%)	0.06
	Moderate	12 (16.9%)	9 (6.5%)	
	Severe	2 (2.8%)	4 (2.9%)	
**1-month post-TAVI**				
AV peak velocity		2.5 ± 1.8	2.2 ± 0.4	0.15
AV mean gradient		10.3 ± 3.8	10.8 ± 4.4	0.5
AVA		1.7 ± 0.42	1.7 ± 0.38	0.9
AVAI		1.09 ± 0.28	1.04 ± 0.25	0.2
LVIDD		47.5 ± 6.7	47 ± 5.5	0.6
Biplane LVEF		58.5 ± 11	60.5 ± 8.7	0.2
LAVI		59.2 ± 25.6	58.4 ± 41.5	0.9
LVMI		118.9 ± 27.5	115.7 ± 26.4	0.4
EE' medial		22 ± 8	22.4 ± 12.3	0.8
EE' lateral		16.7 ± 6.8	16.1 ± 9.2	0.7
S' medial		5.4 ± 1.8	5.4 ± 1.5	0.9
S' lateral		6.7 ± 1.7	6.8 ± 1.9	0.7
LV 2D GLS		−13.6 ± 3.9	−14.3 ± 4.3	0.4
PASP		37.1 ± 9.1	35.3 ± 6.6	0.15
Para-valvular AR	No/Trivial	44 (66.7%)	93 (70.5%)	0.4
	Mild	16 (24.2%)	29 (22%)	
	Moderate	6 (9.1%)	10 (7.6%)	
Prosthetic AV stenosis	Normal	49 (100%)	113 (96.6%)	0.2
	Mild	0 (0%)	4 (3.4%)	
Patient prosthesis mismatch	Insignificant	40 (81.6%)	98 (83.8%)	0.3
	Moderate	9 (18.4%)	15 (12.8%)	
	Severe	0 (0%)	4 (3.4%)	
**1-year post-TAVI**				
AV peak velocity		2.2 ± 0.5	2.3 ± 1.2	0.5
AV mean gradient		10.8 ± 4.2	10.9 ± 5.1	0.9
AVA		1.75 ± 0.48	1.66 ± 0.40	0.2
AVAI		1.13 ± 0.3	1.03 ± 0.26	0.06
LVIDD		44.8 ± 5.7	45.5 ± 5.4	0.5
Biplane LVEF		61 ± 7	61.6 ± 7.6	0.7
LAVI		58.2 ± 29.6	55.3 ± 23.7	0.6
LVMI		105.8 ± 27.7	104.1 ± 23.5	0.7
EE' medial		21.5 ± 7.3	23.3 ± 15.1	0.5
EE' lateral		15.8 ± 6.5	15.3 ± 8.3	0.8
S' medial		5.4 ± 1.3	5.3 ± 1.4	0.6
S' lateral		6.9 ± 1.5	6.8 ± 1.8	0.8
LV 2D GLS		−16.2 ± 3.6	−14.7 ± 4	0.2
PASP		35.8 ± 5.2	34.4 ± 7.2	0.3
Para-valvular AR	No/Trivial	30 (63.8%)	93 (71.8%)	0.3
	Mild	9 (19.1%)	24 (20.5%)	
	Moderate	8 (17%)	9 (7.7%)	
Prosthetic AV stenosis	Normal	36 (94.7%)	93 (93%)	0.7
	Mild	2 (5.3%)	7 (7%)	
Patient prosthesis mismatch	Insignificant	33 (86.8%)	77 (77%)	0.3
	Moderate	5 (13.2%)	18 (18%)	
	Severe	0 (0%)	5 (5%)	

*SIRS, systemic inflammatory response syndrome; AV, aortic valve; AVA, aortic valve area; AVAI, aortic valve area index; LVIDD, left ventricular end-diastolic inner diameter; LVEF, left ventricular ejection fraction; LAVI, left atrial volume index; LVMI, left ventricular mass index; LV 2D GLS, left ventricular two-dimensional global longitudinal strain; PASP, pulmonary artery systolic pressure; RWMA, regional wall motion abnormalities; AR, aortic regurge; MR, mitral regurge; MS, mitral stenosis; TAVI, trans-catheter aortic valve implantation*.

### Predictors of SIRS

A logistic regression model was created for predicting SIRS including potentially relevant laboratory and hemodynamic covariates that showed significant difference on simple comparisons and avoiding any possible multicollinearity (Table 6). On univariate analysis, the pre-procedural Hgb level, serum creatinine, TLC, pre-TAVI heart rate (HR), post-TAVI systolic and diastolic blood pressures, as well as PASP were predictors of SIRS. However, on multiple regression analysis correcting for significant covariates, only pre-TAVI TLC, pre-TAVI HR, and post-TAVI SBP were independent predictors of SIRS.

**Table 6:** Logistic regression analysis for prediction of SIRS.

**Parameter**	**Univariate analysis**			**Multivariate analysis**		
	**OR**	**95% CI**	***P*-value**	**OR**	**95% CI**	***P*-value**
Pre-TAVI TLC	1.3	1.11–1.43	<0.001	1.35	1.10–1.64	0.004
Pre-TAVI creatinine	1.3	1.03–1.52	0.03	1.05	0.82–1.34	0.7
Pre-TAVI Hgb	0.84	0.72–0.98	0.02	0.83	0.65–1.06	0.13
Anesthesia duration	1.0	1.0–1.01	0.06			
Pre-TAVI HR	1.1	1.02–1.07	<0.001	1.05	1.02–1.08	0.003
Pre-TAVI Hs-CRP	1.1	0.97–1.26	0.14			
Post-implant SBP	0.98	0.97–1.0	0.02	0.97	0.95–1.0	0.01
Post-implant DBP	0.98	0.96–1.0	0.046	0.99	0.96–1.03	0.7
Baseline PASP	1.04	1.01–1.07	0.004	1.03	0.99–1.06	0.15

*SIRS, systemic inflammatory response syndrome; OR, odds ratio; CI, confidence interval; TAVI, trans-catheter aortic valve implantation; TLC, total leucocytic count; Hgb, Hemoglobin; HR, heart rate; HS-CRP, high sensitive C reactive protein; SBP, systolic blood pressure; DBP, diastolic blood pressure; PASP, pulmonary artery systolic pressure*.

### Clinical Outcomes

In-hospital and over more than 5-years follow-up clinical outcome data were tracked for patient groups through the hospital electronic medical records. In-hospital clinical outcomes showed that patients with SIRS had higher rates of IH death, cardiac tamponade, major, and minor bleeding episodes, as well as stage I AKI (Table 7). Median follow-up was comparable among both study groups (*p* = 0.4). All-cause death, cardiac death, re-hospitalization for HF, and re-hospitalization for ACS or PCI were significantly more frequent among the SIRS group (Table 7). Looking at the VARC-2 composite endpoints, early safety events (within 30 days), as well as clinical efficacy events (after 30 days), were significantly more frequent among the SIRS group (*p* < 0.001, *p* = 0.009, respectively). On the other hand, device-related events and time-related valve safety were not significantly different between both groups (*p* = 0.6, *p* = 0.2, respectively) (Table 7).

**Table 7:** In-hospital and follow-up clinical outcomes.

**Parameter**			**SIRS (*n* = 80)**		**No SIRS (*n* = 144)**		***P*-value**
**In-hospital**							
Post-TAVI NYHA FC	NYHA 1–2		75 (96.2%)		142 (98.6%)		0.4
	NYHA 3–4		3 (3.8%)		2 (1.4%)		
Transient CAVB			16 (20%)		27(18.8%)		0.8
PPM			6 (7.5%)		11 (7.6%)		0.9
Post-TAVI AF			10 (12.5%)		14 (9.7%)		0.5
Post-TAVI RBBB			7 (8.8%)		17 (11.8%)		0.5
Post-TAVI LBBB			24 (30%)		31 (21.5%)		0.16
IH death			4 (5%)		1 (0.7%)		0.04
Cardiac tamponade			4 (5%)		0 (0%)		0.007
IH Stroke	Ischemic		3 (3.8%)		1 (0.7%)		0.2
	Hemorrhagic		0 (0%)		1 (0.7%)		
IH Bleeding	Minor bleeding	BARC 1	21 (26.3%)	2 (2.5%)	7 (4.9%)	1 (0.7%)	<0.001
		BARC 2		5 (6.3%)		3 (2.1%)	
		BARC 3a		14 (17.5%)		3 (2.1%)	
	Major bleeding	BARC 3b	9 (11.3%)	6 (7.5%)	3 (2.1%)	1 (0.7%)	
		BARC 3c		3 (3.8%)		2 (1.4%)	
IH infective endocarditis			1 (1.3%)		1 (0.7%)		0.7
Access site complication			3 (3.8%)		4 (2.8%)		0.7
Vascular complications	Major		9 (11.25%)	6 (7.5%)	6 (4.2%)	2 (1.4%)	0.06
	Minor			3 (3.8%)		4 (2.8%)	
Aortic dissection			2 (2.5%)		2 (1.4%)		0.5
AKI	Stage 1		11 (13.8%)	10 (12.5%)	4 (2.8%)	3 (2.1%)	0.002
	Stage 3			1 (1.3%)		1 (0.7%)	
**Follow-up**							
Total duration of FU (median, IQR)			610.5 (202–1,110)		682.5 (364–1,225)		0.4
Cardiac death			6 (7.5%)		1 (0.7%)		0.005
All-cause death			11 (13.8%)		2 (1.4%)		<0.001
Re-hospitalization for HF			7 (8.8%)		4 (2.8%)		0.047
Re-hospitalization for ACS/PCI			5 (6.3%)		2 (1.4%)		0.045
Stroke	Ischemic		1 (1.3%)		2 (1.4%)		0.6
	Hemorrhagic		0 (0%)		2 (1.4%)		
**VARC-2 composite endpoints**							
Device-related events			17 (21.3%)		26 (18.1%)		0.6
Early safety (within 30 days)			16 (20%)		7 (4.9%)		<0.001
Clinical efficacy (after 30 days)			11 (13.8%)		6 (4.2%)		0.009
Time-related valve safety			17 (21.3%)		21 (14.6%)		0.2

*SIRS, systemic inflammatory response syndrome; TAVI, trans-catheter aortic valve implantation; NYHA FC, New-York heart association functional classification; CAVB, complete atrioventricular block; AF, atrial fibrillation; RBBB, right bundle branch block; LBBB, left bundle branch block; PPM, permanent pacemaker; IH, in-hospital; BARC, Bleeding academic research consortium; AKI, acute kidney injury; FU, follow-up; IQR, inter-quartile range; HF, heart failure; ACS, acute coronary syndrome; PCI, percutaneous coronary intervention; VARC, Valve academic research consortium*.

Cox-regression analyses for time-adjusted events were displayed for the outcomes of all-cause mortality, and composite endpoint of clinical efficacy (Table 8). Multiple analysis showed that for the outcome of all-cause mortality, STS score (HR = 1.1, 95% CI = 1.01–1.18, *p* = 0.02), Pre-TAVI Hs CRP (HR = 1.3, 95% CI = 1.05–1.51, *p* = 0.01), and post-TAVI peak CK-MB (HR = 1.0, 95% CI = 1.0–1.09, *p* = 0.03) independently predicted all-cause mortality. For the outcome of clinical efficacy, STS score was the only independent predictor (HR = 1.06, 95% CI = 1.03–1.09, *p* < 0.001). Of note, although SIRS showed almost three times the hazard of predicting events for the outcome of clinical efficacy, yet it only showed marginal significance (HR = 2.8, 95% CI = 0.93–8.6, *p* = 0.06).

**Table 8:** Cox regression analysis for the predictors of overall mortality and clinical efficacy.

**Parameter**	**Univariate analysis**			**Multivariate analysis**		
	**HR**	**95% CI**	***P*-value**	**HR**	**95% CI**	***P*-value**
**All-cause mortality**						
Pre-TAVI LVEF	0.94	0.91–0.98	0.001			
Pre-TAVI PASP	1.0	0.99–1.09	0.2			
STS score	1.1	1.05–1.1	<0.001	1.1	1.01–1.18	0.02
Pre-TAVI Hgb	0.8	0.65–0.98	0.03			
Post-TAVI Hgb	0.7	0.49–1.12	0.2			
IH Major bleeding	7.5	1.94–28.68	0.003			
Pre-TAVI TLC	1.2	1.13–1.38	<0.001			
Post-TAVI TLC	1.1	0.99–1.23	0.07			
Pre-TAVI creatinine	1.3	1.08–1.56	0.005			
Post-TAVI creatinine	1.3	1.04–1.61	0.02			
AKI	4.1	1.12–14.85	0.03			
Pre-TAVI Hs-CRP	1.3	1.14–1.48	<0.001	1.3	1.05–1.51	0.01
Post-TAVI peak Hs-CRP	1.2	1.09–1.22	<0.001	1.1	0.99–1.17	0.07
Post-TAVI peak CK-MB	1.0	1.0–1.07	0.04	1.0	1.0–1.09	0.03
Post-TAVI peak Tn	1.1	1.03–1.13	0.002			
SIRS	10.5	2.32–47.33	0.002	6.4	0.67–61.1	0.1
**Clinical efficacy**						
Pre-TAVI LVEF	0.96	0.92–0.99	0.01			
Pre-TAVI PASP	1.0	0.96–1.06	0.7			
STS score	1.1	1.05–1.09	<0.001	1.06	1.03–1.09	<0.001
Pre-TAVI Hgb	0.9	0.73–1.16	0.5			
Post-TAVI Hgb	0.9	0.63–1.22	0.4			
IH Major bleeding	3.8	0.84–17.49	0.08			
Pre-TAVI TLC	1.2	1.09–1.36	0.001			
Post-TAVI TLC	1.0	0.91–1.18	0.6			
Pre-TAVI creatinine	1.2	1.01–1.48	0.04			
Post-TAVI creatinine	1.2	0.96–1.51	0.1			
AKI	1.8	0.41–7.91	0.4			
Pre-TAVI Hs-CRP	1.1	0.93–1.35	0.2			
Post-TAVI peak Hs-CRP	1.1	1.03–1.16	0.002			
Post-TAVI peak CK-MB	1.0	0.96–1.05	0.8			
Post-TAVI peak Tn	1.0	0.98–1.12	0.2			
SIRS	3.4	1.26–9.26	0.016	2.8	0.93–8.6	0.06

*HR, hazard ratio; CI, confidence interval; LVEF, left ventricular ejection fraction; PASP, pulmonary artery systolic pressure; STS, society of thoracic surgeons; Hgb, hemoglobin; IH, in-hospital; TAVI, trans-catheter aortic valve implantation; TLC, total leucocytic count; AKI, acute kidney injury; HS CRP, high sensitive C reactive protein; CK-MB, creatine kinase- myocardial band; Tn, troponin; SIRS, systemic inflammatory response syndrome*.

**Figure 2:**
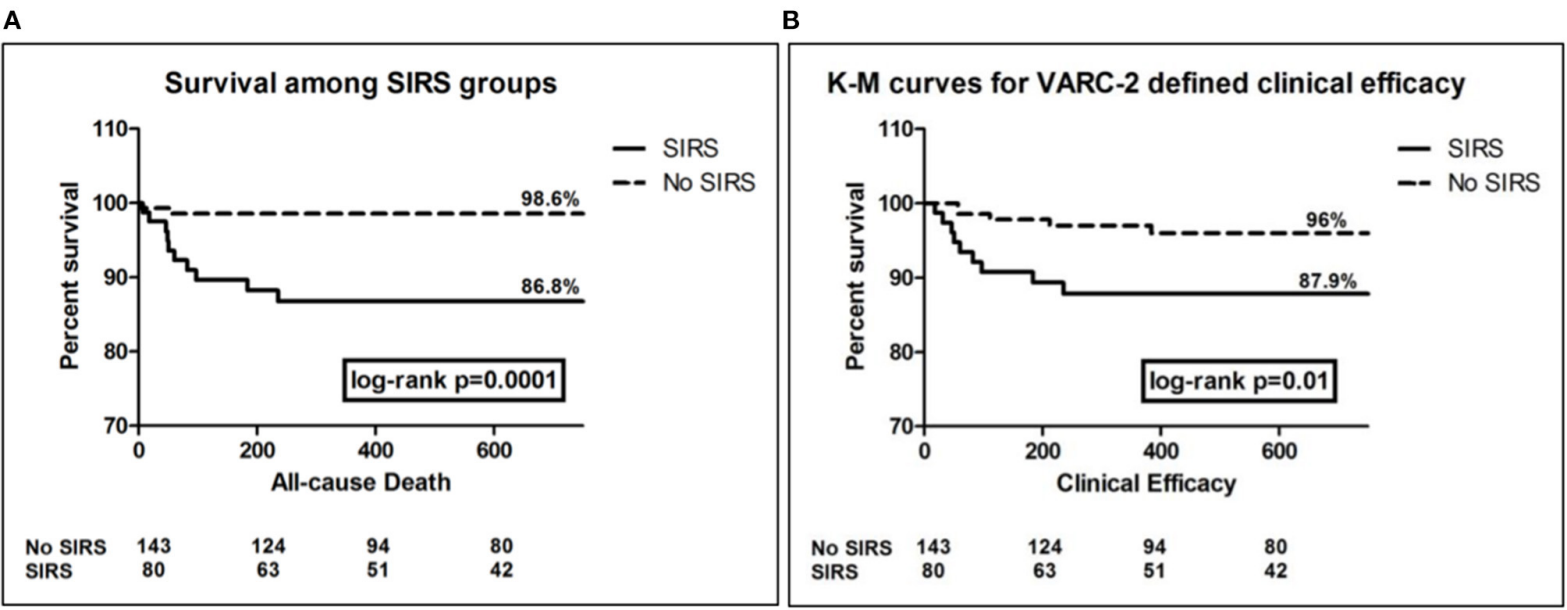
Kaplan Meier survival curves for the outcomes of all-cause death **(A)**, and clinical efficacy composite end-point **(B)**, showing significantly more events among SIRS group, log rank *p* = 0.0001, and 0.01, respectively.

Furthermore, elaborating on survival function of both study groups using K-M analysis, showed that SIRS group had more frequent time-related events for all-cause mortality as well as for the composite endpoint of clinical efficacy (log-rank *p* < 0.001, and =0.01, respectively) (Figure 2).

## Discussion

With the growing application of TAVI in a wide spectrum of aortic stenosis patients ranging from low to high risk, there have been concerns regarding occurrence of SIRS which in turn impacts resource utilization and patient outcomes.

In our study, SIRS has been recorded in 36% of the patient population. This was concordant with the prevalence reported by Schwietz et al. (18) (39.1%), and Sinning et al. (7) (40.1%), confirming the wide prevalence of post-TAVI inflammatory response. This incidence was as high as 73% in a study that included patients undergoing aortic valve replacement via TAVR (*n* = 264) or SAVR (*n* = 483), using an alternative non-femoral access approach in more than half the cases of TAVR, and trans-femoral cases were performed with a surgical cut-down (19).

In our study, pre- and post-procedural markers of inflammation including HS-CRP were significantly higher among the SIRS group. This is in line with the study by Sinning et al. (7), which showed significant increase in pro-inflammatory cytokines (IL-6 and IL-8), with subsequent elevation in acute phase reactants CRP and PCT.

### What Causes SIRS in TAVI Patients?

Among different parameters that were included in a prediction model for the outcome of SIRS, it was concluded that lower post-procedural BP, among other parameters, independently predicted the occurrence of SIRS. It has been previously demonstrated that systemic hypotension results in suboptimal tissue perfusion with subsequent ischemia/reperfusion injury, which in turn triggers a vicious circle of leucocyte and endothelial activation, cytokine release, and finally SIRS (20–22).

It has been postulated that general anesthesia might increase the incidence of SIRS after TAVI (19). However, general anesthesia was employed in the majority of our patient population with no significant difference between both patient groups (*p* = 0.4). Not to mention that the incidence of SIRS was more or less similar to that reported by Sinning et al. (7), in whom TAVI was performed without general anesthesia (conscious sedation). Nevertheless, in our study population, the duration of anesthesia was significantly longer among the SIRS group, which supports the notion that cardiac hemodynamic challenges, more frequently encountered with longer anesthesia duration, is a major determinant in the pathogenesis of SIRS.

Our study provides the largest patient cohort assessing the impact of various valve types of different generations on the incidence of SIRS. Prior studies have assessed the incidence of SIRS either utilizing only self-expanding valves (Medtronic CoreValve®) (7) or balloon-expandable valves (Edwards SAPIEN®) (19). One might expect that shear stress (23), and transient organ hypoperfusion resulting from rapid pacing with the deployment of balloon-expandable valves (19) or, on the other hand, the hemodynamic compromise with slow deployment or recapturing of self-expandable valves (7), might favor one type of valve or another. However, our data showed that there was no difference between valve types among both groups.

### Impact of SIRS on Echocardiographic Findings

Previous study showed that higher leucocytic count and maximum CRP levels post-TAVI were independent predictors of declined LVEF (3). On the contrary, serial echocardiographic assessments of patients in our study, at different time intervals, did not show difference in LV function recovery between both groups, including LV subclinical dysfunction assessed by speckle-tracking derived LV-GLS. This might be explained by the overwhelming beneficial influence of the valve implantation and relief of the LV overload which supersedes the potential impact of any post-procedural inflammatory status. In fact, an early (1 week), kind of paradoxical, more rapid recovery of LV-GLS was noticed among the SIRS group, with a later catch-up at the subsequent time intervals (1 month, 1 year). A recent study, evaluating circulating inflammatory T-cell phenotypes and its association with adverse LV remodeling post-TAVI, assumed a possible role of IL-10, produced by T-cells, as an anti-inflammatory mediator, which improves cardiac function via activating fibroblasts which has a beneficial effect in the early injury phase, whereas long-term chronic activation of fibroblasts increases myocardial fibrosis resulting in adverse remodeling and functional decline (24). This hypothesis-generating assumption should be elaborated in further dedicated larger scale studies.

### Impact of SIRS on Follow-Up CT Findings

For the first time, we assessed the possible relation between post-TAVI SIRS and CT findings on follow-up, particularly the intriguing finding of HALT. Several hypotheses have been postulated in the pathogenesis of HALT (25–28). An inflammatory hypothesis has been implicated in the provocation of HALT (6), which in turn was suspected to trigger inflammatory process with subsequent valve leaflet degeneration (29).

We presumed that subclinical leaflet thrombosis or CT finding of HALT might be partly due to an ongoing inflammatory status that has been early manifested as SIRS. Hypothetically, a sort of chronic activation of systemic inflammatory response might contribute to this delayed finding, and SIRS might be an early trigger. Likewise, inflammatory cascades have been implicated in atherothrombotic coronary disorders and this led to several late therapeutic intervention studies aiming to abort this inflammatory milieu (30, 31). Despite the incidence of HALT in our study was more frequent in the SIRS group of patients (31 vs. 20%), yet this wasn't statistically significant. It is noteworthy that the rate of follow-up CT among SIRS group was less than that of non-SIRS group, thus underestimating the outcome.

### Impact of SIRS on Clinical Outcomes

Previous studies have elucidated increased 30-days (7, 19), and 1-year mortality (7, 18, 19) among TAVI patients eliciting ≥2 SIRS criteria. We found a higher incidence of in-hospital mortality, major and minor bleeding, and acute kidney injury among the SIRS group. Unlike our results, Schwietz et al. showed no significant difference in early mortality, although a significant increase was observed at 1-year (18). The absence of early difference in mortality in their study might be explained by the fact that they employed apical access in almost 38% of their patient population, which entails higher early in-hospital surgical risk.

According to our study, there was a higher incidence of AKI among the SIRS patients, most of them were AKI stage 1. Looking to the baseline patient data, serum creatinine was significantly higher among the SIRS group. However, baseline serum creatinine did not independently predict the occurrence of SIRS. In the study by Sinning et al. (7), SIRS was strongly associated with the development of AKI. Despite the high prevalence of CRF in their series, yet it was not associated with the occurrence of SIRS after TAVI, in concordance with our finding. Previous studies demonstrated that the inflammatory process resulting from ischemia/reperfusion injury plays a role in the pathophysiology of AKI. In TAVI patients, transient peri-procedural renal and gut hypoperfusion might result in significant cytokine release expressed as SIRS, which plays a crucial role in the pathogenesis of AKI (32, 33).

Noticeably, in our study, the incidence of major and minor bleeding was significantly higher in the SIRS group. One of the proposed contributing factors to the development of SIRS in TAVI patients was transfusion of red blood cell (RBC) units (7). This has been previously elucidated in cardiac surgery patients, and was attributed to co-administration of inflammatory mediators e.g., IL-8 which accumulates in the stored packed RBCs (34, 35). However, in our study, baseline Hgb level did not independently predict the occurrence of SIRS in a multivariable regression model. Likewise, Sinning et al. showed that, in their study cohort, the amount of RBC transfusions as well as the net drop in Hgb level didn't predict SIRS (7). This emphasizes that bleeding and subsequent blood transfusion was only a minor contributor to the occurrence of SIRS.

Over more than 5 years median follow-up, SIRS patients had significantly higher rates of overall mortality, cardiac mortality, readmissions due to HF or due to ACS/PCI. Adopting the VARC-2 composite endpoints, SIRS patients did significantly worse in terms of early safety (<30d) and late clinical efficacy (>30d), yet device success rates and time-related valve safety were not significantly different.

Our study provides the longest follow-up for the impact of SIRS on VARC-2-defined individual and composite clinical outcomes, among a real-world population with variable risk and utilizing earlier and latest generations transcatheter aortic valves. Survival analysis for all-cause mortality and VARC-2 composite endpoint of clinical efficacy showed significantly more events among the SIRS patients. This goes along with previous studies (7, 19), which provided survival analysis for 1-year only. In accordance to Sinning et al. (7), who showed a hazard ratio (95% CI) = 7.4 (3.5–15.6) among SIRS group of patients for the outcome of all-cause mortality, our SIRS group showed a hazard ratio (95% CI) = 10.5 (2.32–47.33). However, adjusted for other covariates, SIRS did not independently predict mortality. Despite SIRS showed almost 3 times the hazard of events for the composite outcome of clinical efficacy, yet it failed to independently predict this outcome as well, with only marginal significance (*p* = 0.06).

In summary, TAVI might frequently enhance an inflammatory reaction culminating into SIRS. This happens independent of the valve type, and despite moving toward smaller profile devices with better technological refinements, and a more minimalistic approach (conscious sedation, and more transfemoral procedures with no surgical cut-down), yet SIRS remains a devastating problem which needs all efforts to be attenuated.

### Clinical Implications

Recently, there has been an uprising interest in the concept of inflammation in the pathogenesis of cardiovascular disease (36). Several late trials have tested the efficacy of specific anti-inflammatory medications in the context of atherothrombotic coronary artery disease (30, 31, 37). A recent subgroup analysis of the CANTOS (Canakinumab Anti-inflammatory Thrombosis Outcome Study) trial showed that IL-1β-targeting therapy may decrease heart failure-related hospitalization and mortality (38). Lately, a proof-of-concept study, showed, for the first time, an association of specific inflammatory phenotypes (immune signatures) with increased mortality after TAVI (39). Thus, suggesting that distinct monocyte and T-cell signatures might stand as novel biomarkers to be considered in risk stratifying patients with severe aortic stenosis undergoing TAVI, and possibly guiding potential anti-inflammatory treatment. This remains to be further elucidated in properly designed studies.

## Study Limitations

Owing to relatively small sample size and few events, this study ought to be considered hypothesis-generating. The exact pathophysiological mechanism of SIRS post-TAVI remains speculative, and further studies digging deeper into different proposed triggers for such inflammatory response are warranted.

SIRS criteria are partly determined by vital signs like heart rate, respiratory rate and temperature. One might think that this would entail inaccurate recording with individual bias. However, patients undergoing TAVI were subjected to a standardized, rigorous monitoring protocol in the ICU, where vital signs are recorded in the electronic medical record hourly (or even more frequently if necessitated), and this could be easily tracked and retrieved from the electronic medical system. To be more precise, and to avoid the influence of potentially spurious vital signs on determining SIRS criteria, we included abnormal vitals only when having a minimum of 2 consecutive recordings at least 1 h apart.

Although the study comprises a very small number of patients undergoing TAVI via trans-apical approach, yet the present results might only apply to patients undergoing TAVI using the transfemoral approach.

Finally, despite adjusting for multiple covariates, yet our findings might have been influenced by unmeasured confounders.

## Conclusion

Regardless of valve type, SIRS is frequently encountered after TAVI. It was associated with more frequent in-hospital events, admissions for HF or ACS, as well as long-term mortality. The notion that SIRS might influence the occurrence of HALT is intriguing but couldn't be established. This warrants further research and would impact treatment plans post-TAVI.

## Data Availability Statement

The datasets presented in this article are not readily available because the dataset belongs to Seoul National University Hospital any access to the dataset needs permission from Seoul National University Hospital. Requests to access the datasets should be directed to Hyo-Soo Kim, hyosoo@snu.ac.kr.

## Ethics Statement

The studies involving human participants were reviewed and approved by Seoul National University Hospital Ethics Committee. The patients/participants provided their written informed consent to participate in this study.

## Author Contributions

TA, Y-JK, HE-N, and JK contributed to conception and design of the study. TA, Y-JK, Y-JC, and HE-N organized the database. TA and Y-JK performed the statistical analysis. TA wrote the first draft of the manuscript. All authors contributed to manuscript revision, read, and approved the submitted version.

## Funding

TA received a fully funded post-doctoral scholarship from the Egyptian Ministry of Higher Education and Assiut University.

## Conflict of Interest

The authors declare that the research was conducted in the absence of any commercial or financial relationships that could be construed as a potential conflict of interest.

## Publisher's Note

All claims expressed in this article are solely those of the authors and do not necessarily represent those of their affiliated organizations, or those of the publisher, the editors and the reviewers. Any product that may be evaluated in this article, or claim that may be made by its manufacturer, is not guaranteed or endorsed by the publisher.
