# Supplementary material for: Impact of Systemic Inflammatory Response Syndrome on Clinical, Echocardiographic, and Computed Tomographic Outcomes Among Patients Undergoing Transcatheter Aortic Valve Implantation
Source: Front Cardiovasc Med. 2022 Feb 9;8:746774. doi: 10.3389/fcvm.2021.746774 (PMC8863936; doi:10.3389/fcvm.2021.746774)
Supplement: Supplementary file 1 [file Table_1.docx]

**Supplementary table 1:** Frequency of parameters accounting for the diagnosis of SIRS

| SIRS parameter | Frequency, N (%) |
| --- | --- |
| Temperature >38°C | 35 (43.8%) |
| Temperature <36.0° | 19 (23.8%) |
| Heart rate >90 beats/min. | 59 (73.8%) |
| Hyperventilation* | 48 (60%) |
| leucocytic count >12 or <4 (10^9^/L) | 41 (51.2%) |
| Patients having 2 SIRS parameters | 42 (52.5%) |
| Patients having 3 SIRS parameters | 30 (37.5%) |
| Patients having 4 SIRS parameters | 8 (10%) |

*Respiratory rate >20 breaths/min or P_a_CO_2_ <32 mmHg, SIRS: systemic inflammatory response syndrome.
